# Neutral Lipid Metabolism Influences Phospholipid Synthesis and Deacylation in *Saccharomyces cerevisiae*

**DOI:** 10.1371/journal.pone.0049269

**Published:** 2012-11-05

**Authors:** Gabriel Mora, Michael Scharnewski, Martin Fulda

**Affiliations:** Department of Plant Biochemistry, Albrecht-von-Haller Institute, Georg-August University Goettingen, Goettingen, Germany; Simon Fraser University, Canada

## Abstract

Establishment and maintenance of equilibrium in the fatty acid (FA) composition of phospholipids (PL) requires both regulation of the substrate available for PL synthesis (the acyl-CoA pool) and extensive PL turnover and acyl editing. In the present study, we utilize acyl-CoA synthetase (ACS) deficient cells, unable to recycle FA derived from lipid deacylation, to evaluate the role of several enzymatic activities in FA trafficking and PL homeostasis in *Saccharomyces cerevisiae*. The data presented show that phospholipases B are not contributing to constitutive PL deacylation and are therefore unlikely to be involved in PL remodeling. In contrast, the enzymes of neutral lipid (NL) synthesis and mobilization are central mediators of FA trafficking. The phospholipid:DAG acyltransferase (PDAT) Lro1p has a substantial effect on FA release and on PL equilibrium, emerging as an important mediator in PL remodeling. The acyl-CoA dependent biosynthetic activities of NL metabolism are also involved in PL homeostasis through active modulation of the substrate available for PL synthesis. In addition TAG mobilization makes an important contribution, especially in cells from stationary phase, to FA availability. Beyond its well-established role in the formation of a storage pool, NL metabolism could play a crucial role as a mechanism to uncouple the pools of PL and acyl-CoAs from each other and thereby to allow independent regulation of each one.

## Introduction

The pathways for *de novo* PL synthesis are well understood [1], [2], and also most of the complex mechanisms that regulate the flow of metabolites through phosphatidic acid (PA) into the synthesis of different PL classes are elucidated [3], [4]. On the other hand, regulation of the PL acyl chain composition still holds wide open questions.

The acyl chain composition differs substantially between different lipid classes and it is assumed that regulation is executed during *de novo* synthesis as well as through acyl editing on existing lipid molecules. The existence of alternative pathways both for the synthesis of PA and of the major membrane components phosphatidylcholine (PC) and phosphatidylethanolamine (PE) allows for some species flexibility of *de novo* PL synthesis [5]–[8]. However, the FA specificity of the enzymes involved in the biosynthesis is not sufficient and as an additional parameter the regulation of the acyl-CoA pool composition might be required. This is in part accomplished through control of *de novo* FA synthesis [9], [10]. But, since additional sources feed into the acyl-CoA pool, complementary mechanisms of control must be in place. In recent years the enzymes and the pathways involved in the synthesis and degradation of triacylglycerol (TAG) and steryl esters (SE) have been identified and links between NL metabolism and other cellular processes have been established [11]. It now begins to become clear that NL metabolism plays a central role within the mechanisms regulating the acyl-CoA pool [12], [13] and can therefore have a direct incidence on PL synthesis. In *S. cerevisiae* TAG is synthesized from diacylglycerol (DAG) in either an acyl-CoA independent reaction catalyzed by the PDAT Lro1p [14], [15] or in an acyl-CoA dependent reaction mainly catalyzed by Dga1p [16]–[18]. The enzymes Are1p and Are2p also make a minor contribution to acyl-CoA dependent TAG synthesis [17]; their main role, however, is the synthesis of SE [19], [20]. TAG mobilization on the other hand, producing DAG and a free fatty acid (FFA), is mediated by the lipid particle lipases Tgl3p, Tgl4p and Tgl5p [21], [22] and the mitochondrial lipase Tgl2p [23]. Tgl2p and Tgl3p are also capable of deacylating DAG [23], [24], forming monoacylglycerol which can then be further degraded by the lipid particle lipase Yju3p [25]. In addition, Tgl3p, Tgl4p and Tgl5p have lysophospholipid acyl transferase activities independent of their lipase activities [26], [27]. SE hydrolysis, releasing a FFA and a sterol molecule, depends on the proteins Yeh1p, Yeh2p and Tgl1p [28]–[30].

*De novo* PL synthesis, however, is not enough to account for the actual acyl chain composition of lipids in eukaryotic cells [31]. It is well established that acyl editing occurs extensively and that it is of remarkable importance to the formation of the steady state lipid profile [32]. While substantial advance has been achieved in recent years towards understanding the reacylation step of lipid remodeling [33]–[39], deacylation, the first step, remains obscure. This reaction is expected to follow a phospholipase A- or B- like mechanism but, in yeast, clear evidence for the involvement of particular lipases in the process is extremely scarce. The role of Cld1p in the remodeling of cardiolipin [40] is perhaps the only fully established case. For the major phospholipids, PC, PE, phosphatidylserine and phosphatidylinositol (PI), the spectra of known phospholipases A or B that could mediate remodeling is rather small. Four phospholipases B have been identified in *S. cerevisiae*: Plb1p, Plb2p, Plb3p [41]–[44] and Nte1p [45], [46], all of which possess lysophospholipase in addition to phospholipase B activity. Known Phospholipases A are even more scarce. Lro1p possesses phospholipase A activity and is therefore a candidate to mediate PL remodeling; however, the lipase activity of Lro1p is combined with acyltransferase activity, resulting in the withdrawal of an acyl chain from the *sn-*2 position of PC or PE and its transfer onto DAG, forming TAG rather than releasing a FFA [14], [15]. Altogether, the recent discoveries revealed a complex network of enzymatic activities which is obviously mediating the exchange of acyl chains between neutral lipids and phospholipids. Although it has not yet been shown in detail, it can be assumed that this network is directly reflecting on the actual acyl chain composition of phospholipids.

Experimentally, the contribution of the different processes or proteins to lipid homeostasis, and in particular to PL remodeling, is most commonly addressed through evaluation of the resulting lipid species. Analysis of the intermediaries is enormously challenging, if not, in some cases, wholly unviable, given that lysolipids and FFA are rapidly processed either by degradation or reacylation. In the case of FA, metabolization must be preceded by activation, a reaction for which five ACS are available in *S. cerevisiae* [47], [48]. Deletion of the genes coding for at least two of these enzymes (*FAA1* and *FAA4*) prevents FA recycling and leads to a very strong accumulation of FFA derived from lipid deacylation [49], [50]. In mutant cells lacking both activities the released FFA can neither be reused for lipid synthesis nor be degraded by β-oxidation. The FFA accumulate in cells and medium and we showed previously that their concentration can be considered as a direct measure of lipid remodeling and lipid degradation [49]. Such mutant strains provide an experimental system in which lipid turnover, PL remodeling and FA homeostasis in general can be analyzed from a new perspective.

**Figure 1:**
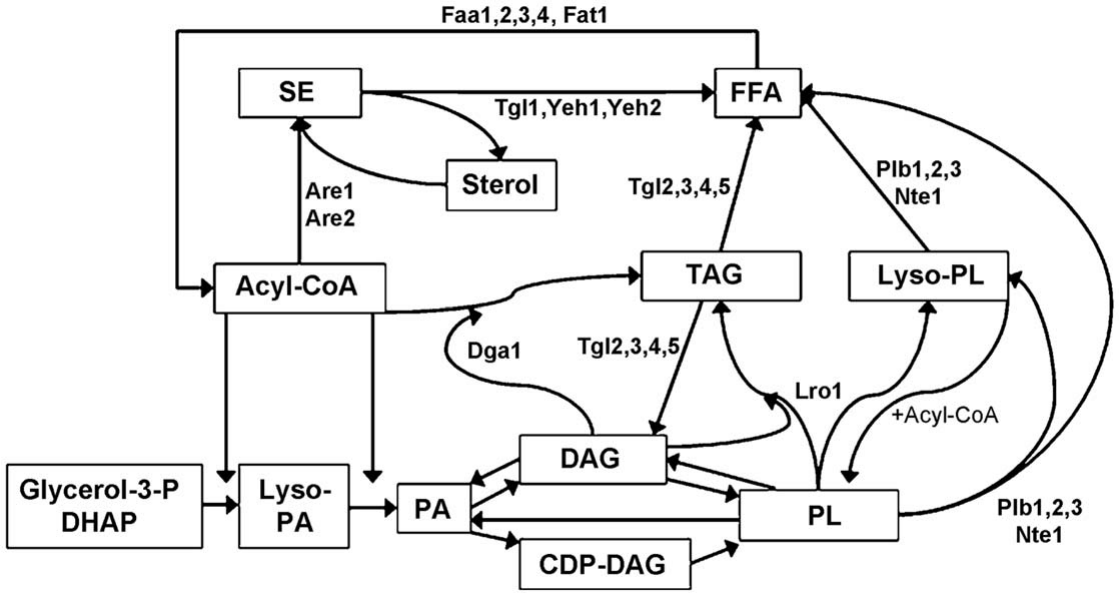
Overview of lipid metabolism in yeast and the enzymes deleted within this work. Formation of lyso-PA from dihydroxyacetone phosphate (DHAP) is mediated by synthesis and reduction of 1-acyl-DHAP. Compounds in alphabetic order: Acyl-CoA, acyl-coenzymeA; CDP-DAG, cytidinediphosphate-diacylglycerol; DAG, diacylglycerol; DHAP, dihydroxyacetone phosphate; FFA, free fatty acid; Glycerol-3-P, glycerol-3-phosphate; Lyso-PA, lyso-phosphatidic acid; Lyso-PL, lyso-phospholipid; PA, phosphatidic acid; PL, phospholipid; SE, steryl ester; TAG, triacylglycerol. Enzymatic activities in alphabetic order: Are1,2, acyl-CoA:sterol acyltransferase 1 and 2; Dga1, diacylglycerol acyltransferase 1; Faa1,2,3,4 and Fat1, fatty acid activation 1 to 4 and fatty acid transporter 1 (acyl-CoA synthetases); Lro1, phospholipid:diacylglycerol acyltransferase (PDAT); Nte1, phosphatidylcholine phospholipase B; Plb1,2,3, phospholipase B 1 to 3; Tgl1,Yeh1,Yeh2, steryl ester hydrolases; Tgl2,3,4,5, triacylglycerol lipase 2 to 5.

In the present study we utilize the ACS deficient strain YB526 (YB332*faa1Δfaa2Δfaa3Δfaa4Δ*) as a FFA accumulating background. Deletion of the genes coding for phospholipases B and enzymes of NL synthesis and degradation on this background reveals their contribution to cellular FA traffic and allows for a discussion on their role in PL and acyl-CoA homeostasis (Fig. 1).

## Results

As has been described before [51], cells carrying deletions in both *FAA*1 and *FAA4* are flocculent in liquid culture, making the determination of cell densities extremely difficult and unreliable. The replacement of glucose as carbon source by raffinose restores homogenously disperse growth [49] and was therefore used in the following experiments. In all cases the optical density at 600 nm (OD_600_) was used as an indicator of cell density. Independence between the genetic composition of the strains and the cell density to OD_600_ ratio is demonstrated in Figure S1. The various mutant strains presented very similar growth rates and saturation densities (Table S1) as well as comparable levels of survival after prolonged starvation (Figure S2). Wild type cells contained only very small amounts of FFA (0.3 and 0.8 µmol/L·OD_600_ at the end of exponential growth and late stationary phase, respectively). Absence of the acyl-CoA synthetases Faa1p, Faa2p, Faa3p and Faa4p led to a dramatic increase in the FFA content (34.2 and 69.9 µmol/L·OD_600_ at the end of exponential phase and late stationary phase, respectively). ACS deficient mutants accumulated FFA intracellularly through all culture stages. Additionally, this phenotype was accompanied by FFA secretion through exponential growth and re-import into the cells upon entrance to stationary phase. Since it was demonstrated that the majority if not all the FA of both pools were once esterified to lipid molecules, they can also be regarded as one pool of released FA. To reflect this view, the results presented here correspond to total FFA at the time of sampling and no further distinction between intra- and extracellular FFA is made (for a detailed description of FFA accumulation progression through different culture stages and the export/import relation see [49]).

**Figure 2:**
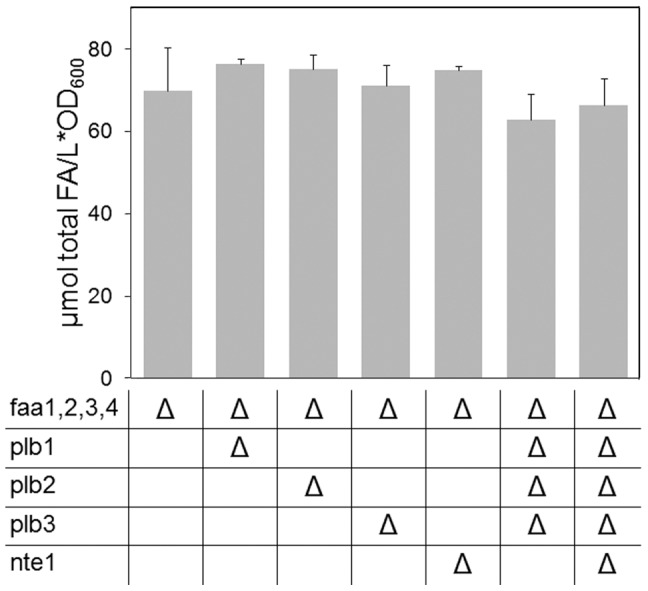
FFA content in phospholipases B deficient mutant strains. Presented is a comparison of the total (sum of 14∶0 to 18∶1) FFA in YB526 (ACS deficient) cells and YB526 cells additionally deficient for phospholipases B. The genotypes of the individual mutant strains are indicated in this and the following figures in table format below the bars. Δ is indicating the deletion of the respective gene and several Δ in one column indicate the combined deletion of the respective genes in one strain. The last column, for example, is referring to the eight-fold mutant deficient of all FAA genes and all phospholipases B. The values presented for FFA in this and the following figures include both FFA retained in the cells and secreted to the media. The FA species composition corresponding to these results and those in Figures 3, 5, 6 and 7 are presented in Table S2. Cells were grown to late stationary phase (136 h) in YPR media. Mean values of at least three independent experiments. Error bars correspond to standard deviation.

As a first step to elucidate the activities involved in the release of the observed FFA, single deletions of the genes coding for any of the phospholipases B (Plb1p, Plb2p, Plb3p and Nte1p), as well as simultaneous deletion of all four genes, were introduced into the YB526 strain. Remarkably, these mutations had no effect on the extent of FFA formation (Fig. 2). These results clearly indicate that these phospholipases B are not required for lipid turnover and in fact suggest that, at least under our experimental conditions, their contribution to basal phospholipid deacylation is negligible.

**Figure 3:**
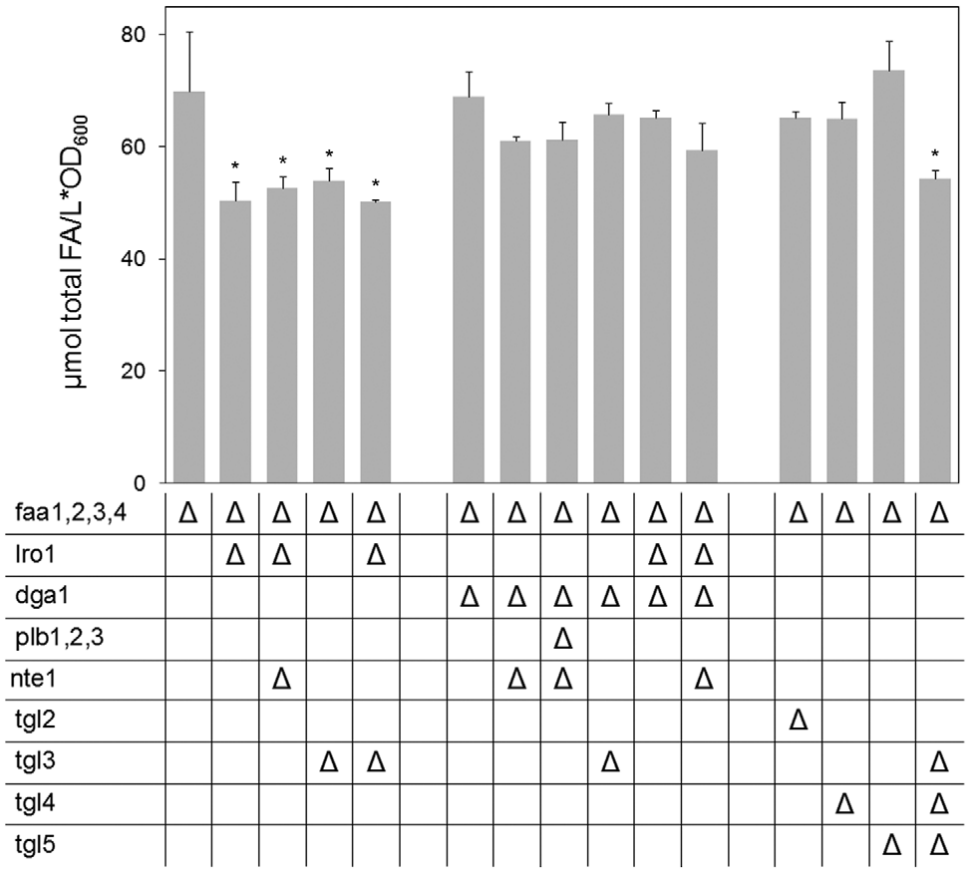
Impact of TAG metabolism on FFA homeostasis. Given are the content of total FFA in YB526 cells and YB526 cells additionally deficient for combinations of the DAG acyltransferases *LRO1* and *DGA1*, TAG lipases and phospholipases B. Cells were grown to late stationary phase (136 h) in YPR media. Mean values of at least three independent experiments. Error bars correspond to standard deviation. Asterisks indicate significantly different values between the reference strain YB526 and the individual mutant strain (P≤0.05).

Along with the phospholipases B, the PDAT Lro1p has been considered as a potential mediator of PL remodeling. Deletion of *LRO1* in our ACS deficient system resulted in a considerably reduced FFA pool (reduction of FFA by 28%, Fig. 3). Given that Lro1p does not release FFA, this decrease must be an indirect effect, most likely reflecting the interrupted synthesis of Lro1p products and their subsequent degradation. The activity of Lro1p results in the synthesis of both lysolipids and TAG; however, out of these two products only the degradation of TAG appears to be of relevance for FFA formation. Zhang and coworkers [52] have shown that lysolipid degradation requires the phospholipase B proteins. Since our *PLB* deletion mutants showed no reduction in FFA content, it can be assumed that the lysolipids produced by Lro1p are reacylated rather than degraded and, therefore, their interrupted synthesis cannot be the reason for the FFA decrease in *LRO1* deficient cells. To evaluate the contribution of TAG degradation to FFA formation, single and multiple deletions of TAG lipases were introduced into the YB526 strain. Deletion of *TGL3* caused approximately the same decrease in the FFA pool as the deletion of *LRO1* (23%, Fig. 3). Deletion of only *TGL2, TGL4* or *TGL5* had no significant effect and the strain YB526*tgl3Δtgl4Δtgl5Δ* resulted in the same FFA content as YB526*tgl3Δ* (Fig. 3), indicating that under our experimental conditions FFA release from TAG is mainly mediated by Tgl3p.

To test the idea that the reduction of the FFA pool seen in the *LRO1* deletion mutant corresponds to a reduction in constitutive TAG degradation, a YB526*lro1Δtgl3Δ* mutant was generated. Outstandingly, the effect of the deletions was not even partially additive. This strain contained the same amount of FFA as either of the mutants lacking only one of the genes (Fig. 3), indicating that no significant amount of FA was constitutively released by Tgl3p in absence of Lro1p.

**Figure 4:**
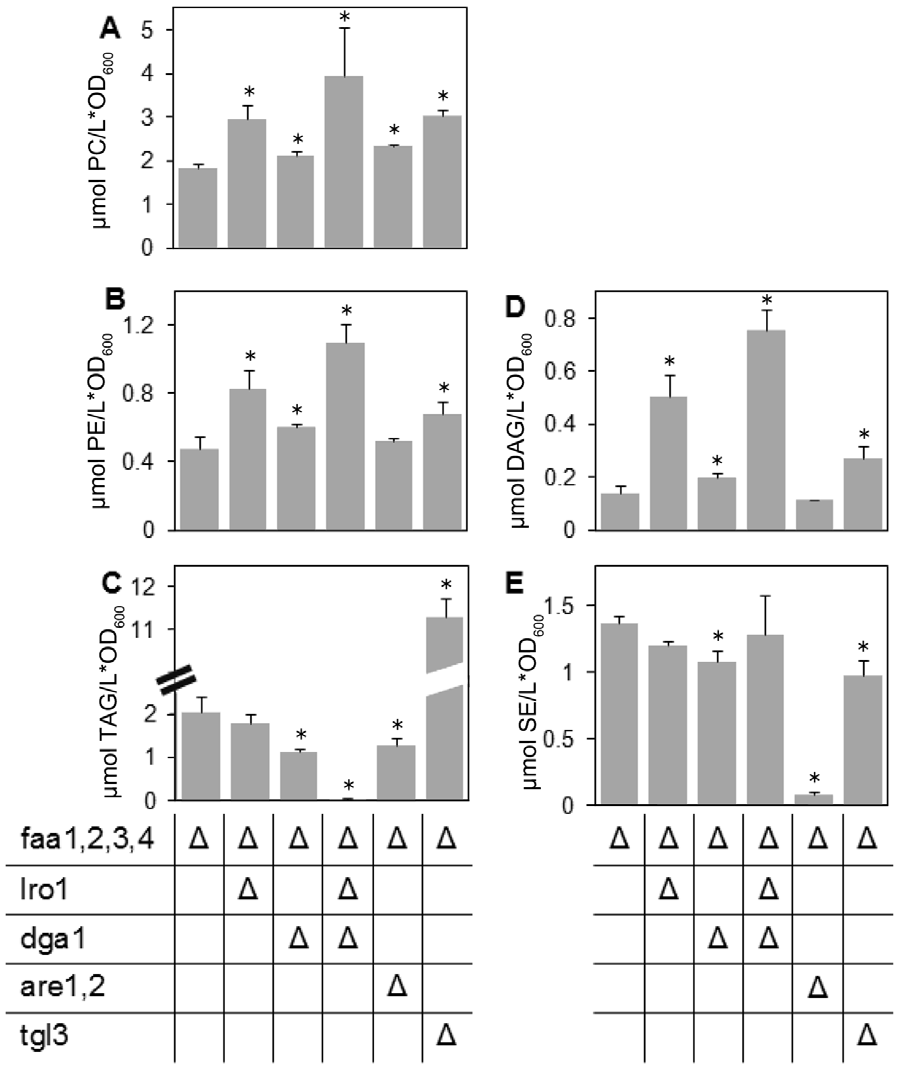
Changes in lipid class composition of mutant strains. Given are the contents of PC (A), PE (B), TAG (C), DAG (D) and SE (E) in YB526 cells and YB526 cells additionally deficient in TAG or SE metabolism. Cells were grown to late stationary phase (136 h) in YPR, lipid classes were separated by TLC and subjected to transmethylation. The resulting FA methyl esters were quantified by GC. Error bars represent the standard deviation in three independent experiments. Asterisks indicate significantly different values between the reference strain YB526 and the individual mutant strain (P≤0.05).

To aid the interpretation of variations in the amount of FFA, quantification of specific lipid classes was carried out for several mutants. No statistically significant decrease of total TAG content was detected in YB526*lro1Δ* cells; however, the relative abundance of 16∶0 in TAG was strongly reduced in this strain. Remarkably, the content of PC, PE and DAG was substantially higher in the YB526*lro1Δ* strain, although the FA profiles of PC and PE showed only minor changes. The profile of DAG, on the other hand, presented an increase in the relative abundance of 16∶0, compatible with the decrease seen in TAG. The strain YB526*tgl3Δ* presented, as expected, a very high TAG content. Surprisingly, elevated amounts of PE and PC as well as an affected FA profile, particularly for PE, were also detected in this strain (Fig. 4 and Table 1).

**Table 1:** Relative FA composition (in percentage) of specific lipid classes.

		16∶0	16∶1	18∶0	18∶1	Total FA
Strain		%				µmol/L•OD
YB526		11 (0.5)	43 (1.1)	9 (0.6)	37 (0.6)	3.64
YB526*lro1Δ*		11 (0.2)	42 (0.9)	8 (0.2)	39 (0.5)	5.91
YB526*dga1Δ*	PC	11 (0.1)	48 (0.8)	8 (0.1)	32 (0.8)	4.25
YB526*lro1Δdga1Δ*		8 (0.1)	45 (0.2)	7 (0.1)	39 (0.1)	7.85
YB526*are1Δare2Δ*		9 (0.5)	44 (0.6)	6 (0.2)	40 (1.1)	4.63
YB526*tgl3Δ*		17 (1.1)	41(0.9)	9 (0.2)	33 (2.3)	6.07
YB526		23 (1.5)	32 (1.1)	2 (0.2)	42 (2.0)	0.95
YB526*lro1Δ*		24 (1.2)	31 (1.9)	2 (0.0)	43 (0.9)	1.65
YB526*dga1Δ*	PE	23 (0.2)	36 (0.4)	3 (0.1)	38 (0.4)	1.21
YB526*lro1Δdga1Δ*		22 (0.4)	33 (0.4)	2 (0.0)	42 (0.0)	2.19
YB526*are1Δare2Δ*		20 (0.8)	30 (0.8)	2 (0.1)	47 (1.5)	1.03
YB526*tgl3Δ*		38 (2.4)	22 (0.5)	5 (0.4)	35 (2.3)	1.35
YB526		14 (0.3)	37 (0.5)	8 (0.1)	41 (0.6)	6.07
YB526*lro1Δ*		10 (0.3)	43 (1.5)	7 (0.4)	39 (0.8)	5.35
YB526*dga1Δ*	TAG	16 (0.6)	33 (0.6)	4 (0.1)	47 (0.3)	3.34
YB526*lro1Δdga1Δ*		14 (0.7)	27 (0.5)	7 (0.1)	52 (0.6)	0.14
YB526*are1Δare2Δ*		9 (0.4)	38 (0.7)	5 (0.3)	46 (1.1)	3.83
YB526*tgl3Δ*		13 (0.7)	32 (0.4)	11 (0.1)	43 (0.8)	33.87
YB526		16 (1.6)	26 (1.3)	8 (1.4)	46 (2.5)	0.28
YB526*lro1Δ*		24 (0.3)	24 (0.9)	6 (0.0)	45 (0.9)	1.01
YB526*dga1Δ*	DAG	17 (0.5)	26 (0.5)	7 (0.1)	42 (1.2)	0.40
YB526*lro1Δdga1Δ*		23 (0.3)	25 (0.4)	6 (0.2)	45 (0.1)	1.51
YB526*are1Δare2Δ*		15 (0.3)	27 (0.5)	7 (0.1)	45 (0.9)	0.23
YB526*tgl3Δ*		21 (1.2)	25 (0.9)	13 (1.0)	41 (1.1)	0.54
YB526		12 (1.4)	41 (5.2)	6 (0.7)	39 (5.6)	1.36
YB526*lro1Δ*		7 (2.2)	33 (2.2)	4 (0.7)	56 (6.5)	1.20
YB526*dga1Δ*	SE	7 (0.8)	32 (1.0)	6 (0.4)	50 (0.8)	1.08
YB526*lro1Δdga1Δ*		4 (1.0)	32 (1.2)	3 (0.1)	59 (1.9)	1.28
YB526*are1Δare2Δ*		18 (1.8)	43 (6.8)	0	39 (8.4)	0.08
YB526*tgl3Δ*		7 (0.7)	31 (2.9)	6 (0.6)	56 (2.9)	0.97

14∶0 is included in the total but is not presented in the table. Cells were grown in YPR to late stationary phase (136 h), lipid classes were separated by TLC and their FA composition determined by GC after transmethylation. The mean values correspond to three independent experiments; standard deviation is shown within parentheses.

To further dissect the role of TAG metabolism in FFA formation a deletion of *DGA1* was introduced in YB526 cells, alone and in combination with the deletion of *LRO1* or *TGL3*. The amount of FFA in the YB526*dga1Δ* strain (Fig. 3) was not lower than in the reference strain. However, the following mutants revealed that, despite the unaffected FFA content, TAG mobilization was altered in these cells. The FFA content in the YB526*tgl3Δdga1Δ* mutant was not lower than in YB526*dga1Δ* (Fig. 3), indicating that no major contribution to FFA formation was mediated by Tgl3p in absence of *DGA1*. However, while the strains YB526*lro1Δ* and YB526*lro1Δtgl3Δ* contained less FFA than the reference strain, YB526*dga1Δ* and YB526*tgl3Δdga1Δ* did not. This suggests that, in absence of *DGA1*, a route independent of TAG synthesis and degradation might come into play resulting in an alternative release of FFA. Similarly, the FFA content in the strain YB526*lro1Δdga1Δ* did not differ from that in the reference strain YB526 (Fig. 3). Since this mutant was almost entirely devoid of TAG (Fig. 4), it is clear that the deletion of *DGA1* led to an enhanced FFA production through a TAG independent pathway.

To investigate if phospholipase activity is involved in the enhanced FFA production through a TAG independent pathway some additional mutant combinations were established. While deletion of *NTE1*, or any of the other phospholipases B, did not affect the amount of FFA in the reference strain, the strains YB526*nte1Δdga1Δ*, YB526*nte1Δlro1Δdga1Δ* and YB526*plb1Δplb2Δplb3Δnte1Δdga1Δ* did present a lower FFA content than YB526*dga1Δ*. This effect did not take place in a YB526*nte1Δlro1Δ* mutant, which had as much FFA as YB526*lro1Δ* (Fig. 3). This suggests that the increased FFA production through TAG-independent mechanisms caused by *DGA1* deletion is at least partly mediated by elevated PL synthesis and degradation.

Further analysis of esterified fatty acids revealed that the situation is more complex than a direct relay of FA from PL or acyl-CoA to TAG to the FFA pool. In contrast to the deletion of *LRO1,* only minor increases in DAG, PC and PE occurred in absence of *DGA1*, however, the FA profiles of PC and PE showed stronger alterations. Interestingly, the effects of deleting *LRO1* or *DGA1* were to some extent opposite: While no significant reduction in TAG content was found upon *LRO1* deletion, a substantial decrease (45%, Fig. 4) resulted from *DGA1* deletion. In YB526*dga1Δ* the TAG profile showed a decrease in the relative abundance of 16∶1 and an increase in the relative abundance of 16∶0 and 18∶1, while the contrary was true for YB525*lro1Δ*. In both PE and PC, the *DGA1-*deficient mutant showed an increase in the relative abundance of 16∶1 and a decrease in 18∶1. The strain YB525*lro1Δ* appeared to show the opposite tendency, although in this case the changes were very small (Fig. 4 and Table 1). In YB526*lro1Δdga1Δ* the increments in the content of DAG, PC and PE were significantly higher than upon deletion of only *LRO1* (Fig. 4).

**Figure 5:**
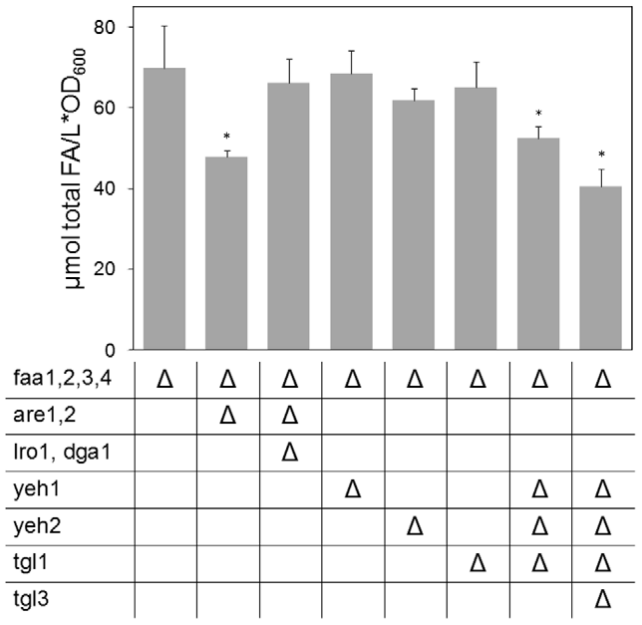
Impact of SE and TAG synthesis and degradation on FFA homeostasis. Displayed are the total FFA in YB526 cells and YB526 cells additionally deficient for enzymes of SE and TAG synthesis and degradation. Cells were grown to late stationary phase (136 h) in YPR media. Mean values of at least three independent experiments. Error bars correspond to standard deviation. Asterisks indicate significantly different values between the reference strain YB526 and the individual mutant strain (P≤0.05).

In a next set of experiments we investigated if SE metabolism also impacts the FFA pool. Our data show that, similar to changes in TAG metabolism, interference with SE metabolism also reflects strongly on the pool of FFA. Inactivation of SE synthesis by simultaneous deletion of *ARE1* and *ARE2* in YB526 reduced the FFA content of the cells by 32% (Fig. 5). As expected, this strain was devoid of SE (Fig. 4). Deletion of *ARE1* and *ARE2* resulted as well in a reduced pool of TAG (37%, Fig. 4) and an increase in PC content (27%, Fig. 4). In the strain YB526 the FA in SE were mainly 16∶1 and 18∶1. Interestingly, the increase in PC upon deletion of *ARE1* and *ARE2* was accompanied by an augmented relative abundance of 16∶1 and 18∶1 (Table 1). Inactivation of the SE degrading enzymes Tgl1p, Yeh1p and Yeh2p, caused a similar decrease in the FFA pool as the abolishment of SE synthesis by deletion of *ARE1* and *ARE2* (25%, Fig. 5).

In the next step, mutants simultaneously deficient in both TAG and SE metabolism were produced. The strain YB526*lro1Δdga1Δare1Δare2Δ*, in which synthesis of TAG and SE is abolished, contained as much FFA as YB526 or YB526*lro1Δdga1Δ* (Fig. 5). The fact that the deletion of *ARE1* and *ARE2* reduced the FFA pool in YB526 cells but not in this strain additionally deficient for *DGA1* and *LRO1*, clearly resembles the situation encountered upon the deletions of *LRO1* or *TGL3*, which affected the FFA content of YB526 cells but not of YB526*dga1Δ* cells (Fig. 3).

Simultaneous deletion of the TAG lipase *TGL3* and the SE hydrolases *YEH1, YEH2* and *TGL1* caused a reduction of the FFA pool by 42% (Fig. 5), roughly corresponding to the sum of the reductions caused by independently blocking TAG or SE mobilization.

**Figure 6:**
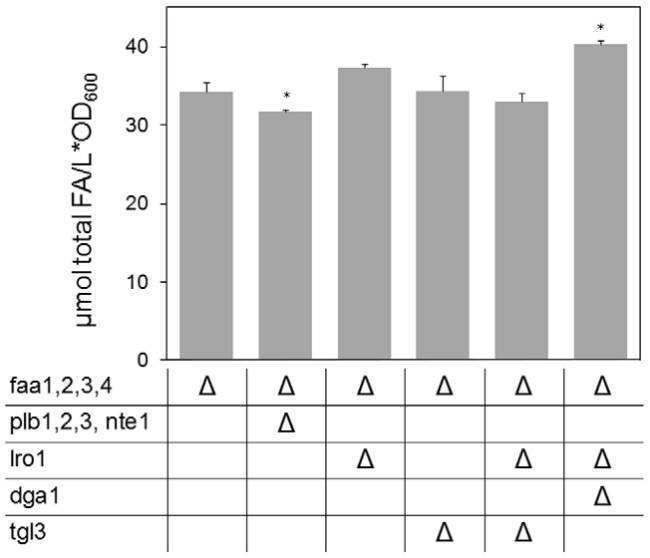
Analysis of specific mutants at the end of exponential phase. Given are the content of total FFA in YB526 cells and YB526 cells additionally deficient for phospholipases B or enzymes of TAG synthesis and degradation. Cells were grown to the end of exponential phase (35 h) in YPR media. Mean values of three independent experiments. Error bars correspond to standard deviation. Asterisks indicate significantly different values between the reference strain YB526 and the individual mutant strain (P≤0.05).

Figures 2 through 5 present results at late stationary phase. Since FFA can at no point leave the system, these measurements constitute a summary of events through all culture stages. However, to differentiate effects arising during exponential and stationary phases, the FFA content of a subset of strains was measured towards the end of exponential growth (Fig. 6). As with the measurement at a later stage, no major change in the size of the FFA pool was encountered upon deletion of all four phospholipases B, ratifying that these enzymes do not play a prominent role in constitutive lipid deacylation.

In contrast to the results at late stationary phase, at this earlier point no reduction of the FFA pool resulted from the deletion of *TGL3* (Fig. 6), indicating that the contribution of Tgl3p mediated TAG mobilization to the formation of the FFA pool occurred mainly in non-dividing cells. At this stage the deletion of *LRO1*, alone or in combination with deletion of *TGL3*, also resulted in no reduction of the FFA pool (Fig. 6). While this is in agreement with the idea that the effect of *LRO1* deletion on FFA formation proceeds through an inhibition of Tgl3p, it leaves the open question whether the role of Lro1p in PL deacylation is of prominence during both exponential and stationary phases or only during the later stage. Remarkably, abrogation of TAG synthesis in the YB526*lro1Δdga1Δ* strain led to an increase in the amount of FFA (18%, Fig. 6). This result is compatible with no contribution of TAG mobilization during exponential phase combined with an increase in TAG-independent FFA formation upon *DGA1* deletion.

**Figure 7:**
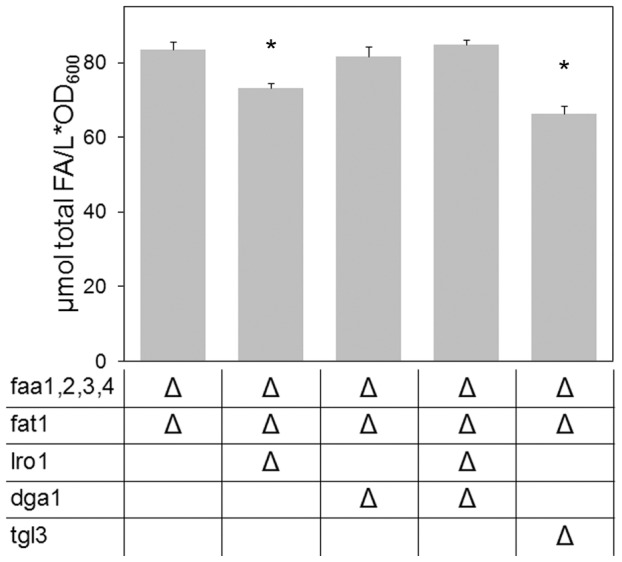
Impact of Fat1p on the FFA pool. Given are the content of total FFA in YB526*fat1Δ* cells and YB526*fat1Δ* cells additionally deficient for enzymes of TAG synthesis and degradation. Cells were grown to late stationary phase (136 h) in YPR media. Mean values of three independent experiments. Error bars correspond to standard deviation. Asterisks indicate significantly different values between the strain YB526*fat1Δ* and the individual mutant strain (P≤0.05).

In the YB526 strain used as a genetic background in this study the ACS Fat1p is still present. This should not affect the interpretation of our results since, in order to evaluate the incidence of a specific deletion on FFA release, we require only that a FFA pool is formed and not that it achieves its maximum possible size. However, to verify the validity of this assumption, *FAT1* was deleted in the strains constituting the core of our analysis. As we have shown before [49], YB526*fat1Δ* cells contain more FFA than YB526. Equivalent to the results on the YB526 background, the FFA pool of YB526*fat1Δ* cells was reduced by the deletions of *LRO1* or *TGL3*, but not by the deletion of *DGA1* or the combined deletions of *DGA1* and *LRO1* (Fig. 7).

## Discussion

While it is a well known fact that lipid turnover is a common process in cells and that it plays a major role in attaining the steady state lipid composition of the cell, the results obtained upon deletion of the acyl-CoA synthetases highlight the actual magnitude of the phenomena: by late stationary phase the cells have turned over an amount of FA corresponding to four times their total content of esterified FA. Given that in WT cells these FA are activated and can reenter lipid metabolism, it also becomes clear that lipid turnover and FA recycling make a major contribution to the acyl-CoA pool in addition to *de novo* FA synthesis. It is also remarkable that the size of the FFA pool doubles between the end of exponential growth and the time point selected at late stationary phase, indicating that lipid metabolism remains highly active in non-dividing cells.

The phospholipases B of yeast have often been mentioned as potential candidates when the need for deacylation is addressed in the context of PL degradation and remodeling [32], [37]. However, under our experimental conditions these enzymes played no substantial role in constitutive lipid deacylation. Similarly, Tanaka and coworkers [53] showed that, upon deletion of *PLB1, PLB2* and *PLB3*, the choline auxotrophic *pem1Δpem2Δ* strain (unable to synthesize PC by methylation of PE) retains its ability to grow in media containing short acyl chain PC (diC_8_PC) as choline source. While their results seem to exempt Plb1p, Plb2p and Plb3p from involvement in the remodeling of an entirely unnatural exogenously provided PL, our results extend this exemption to Nte1p and, most importantly, refer to the endogenous and naturally produced PL content of the cell. These negative results are not entirely surprising since the fact that these proteins possess lysophospholipase in addition to phospholipase B activity does make them suboptimal candidates for the production of lysolipids that should mediate constitutive PL remodeling. In any case, this does not dispute their involvement in non-constitutive processes like glycerophosphodiester formation [54] and lysolipid degradation [41], [42], [52]. Rather than taking part in constitutive remodeling these lipases could mainly degrade excessive phospholipids arising from specific culture conditions, a demonstrated role of phospholipase A enzymes in mammalian cells [55]. In yeast the activity of Nte1p is known to behave in such fashion, resulting in enhanced PC turnover when culture conditions such as inositol supplementation, choline supplementation or elevated temperature lead to an increased rate of PI or PC synthesis [45], [46], [56], [57]. Within our experiments Nte1p, but not the other Plb enzymes, did show a role in PL deacylation upon deletion of *DGA1*, what, as will be discussed ahead, appears to be a situation of increased PL synthesis.

Under our experimental conditions TAG mobilization accounted for almost one quarter of the total FFA produced by the cells and the release was mainly mediated by Tgl3p. Interestingly, this constitutive degradation of TAG was of prominence only during stationary phase, coinciding with the repression of FA synthesis and elongation [10]. Furthermore, the release of FFA by Tgl3p appeared to be strongly linked to the process of TAG synthesis. In terms of the FFA pool size, absence of either *LRO1* or *DGA1* rendered the additional deletion of *TGL3* ineffective, indicating that interference with TAG synthesis prevented its constitutive mobilization. At this stage we can only speculate on how variations on the mode of TAG synthesis could modulate its mobilization. Deletion of *DGA1* resulted in a strong reduction of the TAG pool which should lead to reduced TAG degradation. On the other hand, *LRO1* deficient cells contained a quantitatively unaffected pool of TAG, calling for a different mechanism to affect TAG degradation. The prominent role that Lro1p displayed in FA trafficking speaks strongly in favor of its candidacy as a major mediator of PL remodeling. This view is further strengthened by the direct influence of the deletion on the PC and PE content. Under this circumstances, inhibition of TAG degradation upon absence of *LRO1* would indicate a strong link between PL remodeling and TAG metabolism. In this sense, Rajakumari and coworkers [58] proposed that a certain level of TAG mobilization is specifically directed towards providing FA for PL remodeling. Our results suggest that such mobilization is not only directed towards PL reacylation, but could in fact be controlled by the remodeling process itself. Parallel to this link between PL and TAG deacylation, Horvath and coworkers [59] have shown that a tight relation between PL and TAG synthesis exists as well, overall revealing a profound connection of PL and TAG metabolism. While Horvath and coworkers indicate that the metabolic origin and subcellular localization of the PL, in particular PE, affect the activity of Lro1p, the way in which this activity could modulate TAG degradation remains an open question. Direct regulation of the expression or activity of Tgl3p by the presence of the intermediary lysolipids constitutes an interesting possibility; however, our attempts to demonstrate this relation have so far been unsuccessful (data not shown) and further experimentation will be required to clarify the matter.

Our further mutant strains revealed that Dga1p plays, as well, an important role in the link between PL and TAG metabolism. Despite the important contribution of TAG mobilization to the formation of the FFA pool in YB526 cells, neither impairment in degradation nor the inability to synthesize TAG led to a reduced FFA pool in *DGA1* deficient cells (strains YB526*tgl3Δdga1Δ* and YB526*lro1Δdga1Δ*). This suggests that PL deacylation must be enhanced in absence of *DGA1* (an alternative increase in SE deacylation seems unlikely given the results obtained with YB526*lro1Δdga1Δare1Δare2Δ,* discussed further ahead). However, the content of PE and PC in the strains YB526*dga1Δ* and YB526*lro1Δdga1Δ* was actually elevated. This means that not only degradation but also synthesis of PL was augmented by the deletion of *DGA1*. In line with the hypothesis that *DGA1* deletion led to an elevated rate of PL synthesis and degradation, the YB526*nte1Δdga1Δ* mutant had a lower FFA content than YB526*dga1Δ*. Given that the deletion of *DGA1* prevents the incorporation of large amounts of acyl-CoA into TAG, enhanced PL synthesis could easily be the metabolic consequence. This, however, should have important implications: First, it appears that the excess of acyl-CoA derived from interrupted TAG formation was immediately utilized for lipid synthesis, avoiding the down regulation of FA synthesis or the onset of other mechanisms for the regulation of PL synthesis. Second, the enhanced production then led to increased degradation, indicating that the additional PL resulting from an imbalance of the acyl-CoA pool were not just unnecessary, but might have in fact been inconvenient for the cell. This reveals that Dga1p mediated TAG synthesis serves not only the purpose of producing a stock for later mobilization, but also acts as an active negative modulator of the acyl-CoA pool and, through that, as a modulator of PL synthesis. Petschnigg and coworkers [12] demonstrated that the toxicity of excess FA supplementation, in particular unsaturated FA, is associated with membrane proliferation and an altered species composition of membrane PL due to uncontrolled incorporation of the supplemented FA. Furthermore, these authors, as well as Garbarino and coworkers [60], showed that TAG synthesis acts as a buffer to detoxify the acyl-CoA pool derived from FA import. Our results indicate that, in addition to this role in the regulation of exogenous FA incorporation into PL, Dga1p mediated TAG synthesis serves as well as a mechanism to regulate the incorporation of endogenously produced FA into PL.

While the reduction of the FFA pool in the strain YB526*nte1Δdga1Δ* exposed the phenomena of enhanced PL degradation, it must be noted that the reduction was relatively small (compare with YB526*tgl3Δ*), pointing to the existence of additional/alternative mechanisms for the degradation of the excess PL produced upon *DGA1* deletion. We have identified autophagy as a prominent NL independent pathway for FA trafficking and a major contributor to PL degradation. Autophagy seems to be, on the one hand, responsible for a large portion of the FFA release remaining in cells unable to mobilize NL and, on the other, a major component of the enhanced PL degradation that occurs in response to impaired NL synthesis. Furthermore, contrary to TAG mobilization, autophagy makes an important contribution to FFA formation during both exponential and stationary phases (manuscript in preparation).

As shown by the strain YB526*tgl1Δyeh1Δyeh2Δ*, SE deacylation accounted for another quarter of FFA production, suggesting that SE mobilization does not exclusively occur at times of increased sterol requirement or upon interruption of *de novo* sterol synthesis, but rather that a continuous cycle of SE synthesis and mobilization takes place. This is in agreement with an earlier proposition by Sorger and coworkers [61] recently confirmed by Wagner and coworkers [62] in a report showing that the majority of sterol precursors make transit, as SE, through lipid particles and are then recycled back into the biosynthetic pathway.

The results obtained with the strain YB526*tgl1Δyeh1Δyeh2Δtgl3Δ*, where the FFA pool was reduced by 42%, showed that the contributions of TAG and SE mobilization to FFA formation are essentially additive. In YB526*lro1Δdga1Δare1Δare2Δ*, which can produce neither TAG nor SE, the FFA content was as high as in the background strain YB526, indicating that as in the strains YB526*dga1Δ* and YB526*lro1Δdga1Δ* PL deacylation was enhanced, but to an even greater extent. It is apparent that in this case both the pool of acyl-CoA that could not be utilized for TAG synthesis and the pool that could not be utilized for SE synthesis were incorporated into PL which the cell then needed to degrade. These results clearly support the idea that the ability to synthesize TAG plays a prominent role in modulating the pool of endogenous acyl-CoA in order to make the outcome of PL synthesis match the actual PL requirement of the cell.

In conclusion, our data provide novel insight into the mechanisms of lipid remodeling in yeast. Fatty acid transit through NL metabolism might act as a means to allow rapid regulation of both the PL and the acyl-CoA pools size and acyl chain composition with a higher degree of independence from each other and from *de novo* FA synthesis. PL deacylation through Lro1p instead of a phospholipase A or B can prevent the direct incidence of PL remodeling on the acyl-CoA pool. On the other hand, negative regulation of the endogenous acyl-CoA pool by Dga1p can avoid a direct reflection of *de novo* FA synthesis and lipid degradation on PL synthesis.

## Materials and Methods

### Yeast strains and media

The yeast strains used are shown in Table 2. YPR and YPD consisted of 1% yeast extract, 2% peptone and 2% raffinose or 2% dextrose, respectively. 5-FOA medium consisted of 6.7 g•L^−1^ yeast nitrogen base with ammonium sulfate (MP Biomedicals, Illkirch, France), 0.79 g•L^−1^ complete supplement media (Q-BIOgen, Heidelberg), uracil to 50 mg•L^−1^, 1 g•L^−1^ 5-Fluoro-Orotic Acid and 2% dextrose. 2% agar was added for solid media.

**Table 2:** Yeast strains.

Strain	Genotype	Source
YB332	MATa NMT1 *ura3 his3Δ200 ade2 lys2-801 leu2*	[47]
YB526	YB332 *faa1Δ1.9::HIS3 faa2Δ0.5::LEU2 faa3Δ0.8::LEU2 faa4Δ::LYS2*	[47]
GM26	YB526 *nte1Δ::NAT_loxp_*	This study
GM27	YB526 *lro1Δ*	This study
GM29	YB526 *lro1Δ::KANMX4_loxp_*	This study
GM38	YB526 *lro1Δ dga1Δ::KANMX4*	This study
GM39	YB526 *nte1Δ*	This study
GM46	YB526 *dga1Δ::NAT_loxp_*	This study
GM51	YB526 *are2Δ::HYG are1Δ::NAT_loxp_*	This study
GM60	YB526 *lro1Δ nte1Δ::HYG*	This study
GM61	YB526 *lro1Δ dga1Δ::KANMX4 are2Δ::HYG are1Δ::NAT_loxp_*	This study
GM63	YB526 *tgl3Δ::NAT_loxp_*	This study
GM81	YB526 *tgl3Δ::NAT_loxp_ tgl4Δ::HYG tgl5Δ::KANMX4*	This study
GM103	YB526 *lro1Δ tgl3Δ::HYG*	This study
GM112	YB526 *plb2Δ::NAT_loxP_*	This study
GM113	YB526 *tgl4Δ::NAT_loxP_*	This study
GM114	YB526 *tgl5Δ::NAT_loxP_*	This study
GM115	YB526 *plb1Δ::HYG*	This study
GM116	YB526 *plb3Δ::HYG*	This study
GM120	YB526 *plb2Δ::NAT_loxP_ plb3Δ::HYG plb1Δ::KANMX4*	This study
GM122	YB526 *nte1Δ dga1Δ::NAT_loxp_*	This study
GM123	YB526 *tgl3Δ::NAT_loxp_ dga1Δ::HYG*	This study
GM124	YB526 *lro1Δ dga1Δ::KANMX4 are2Δ::HYG are1Δ*	This study
GM125	YB526 *lro1Δ nte1Δ::HYG dga1Δ::NAT_loxp_*	This study
GM135	YB526 *tgl1Δ::NAT_loxp_*	This study
GM136	YB526 *tgl2Δ::NAT_loxp_*	This study
GM141	YB526 *yeh2Δ:: NAT_loxp_*	This study
GM142	YB526 *yeh1Δ::NAT_loxp_*	This study
GM150	YB526 *yeh2Δ::NAT_loxp_ tgl1Δ::HYG yeh1Δ::KANMX4*	This study
GM153	YB526 *tgl3Δ tgl4Δ::HYG tgl5Δ::KANMX4*	This study
GM164	YB526 *yeh2Δ::NAT_loxp_ tgl1Δ::HYG yeh1Δ::KANMX4 tgl3Δ::URA*	This study
GM180	YB526 *plb2Δ::NAT_loxP_ plb3Δ::HYG plb1Δ::KANMX4 nte1Δ::URA*	This study
GM188	YB526 *plb2Δ::NAT_loxP_ plb3Δ::HYG plb1Δ::KANMX4 nte1Δ::URA dga1Δ::DSDAMX4*	This study
MS51	YB526 *fat1Δ::KANMX4*	[49]
GM190	YB526 *lro1Δ fat1Δ::DSDAMX4*	This study
GM191	YB526 *dga1Δ::NAT_loxp_ fat1Δ::DSDAMX4*	This study
GM192	YB526 *tgl3Δ::NAT_loxp_ fat1Δ::DSDAMX4*	This study
GM194	YB526 *lro1Δ dga1Δ::KANMX4 fat1Δ::NAT_loxP_*	This study

The selection marker used for each deletion is indicated next to the targeted gene. Posterior removal of the marker is indicated by the deleted gene followed by no marker description.

### Cell growth

Yeast cultures were grown for 18 h in YPR and diluted until OD_600_ of 0.03 was reached in triplicate in flasks containing 30 mL of YPR. The cells were grown at 30°C and harvested after 35 hours (end of exponential phase) or 136 hours (late stationary phase). Cell growth was monitored by OD_600_ (Amersham Bioscience, Ultrospec 1100 pro). Cells were harvested by centrifugation and the cell pellet was resuspended in water to achieve appropriate densities (OD_600_ in the range 0.1–0.8). Independence between the strains genotype and the cell number to OD_600_ ratio was verified by counting cells in a subset of late stationary phase cultures. Aliquots were diluted (20× in water) and loaded in a hemocytometer. Cells were counted under a light microscope (Olympus BX51). Cell viability after prolonged starvation was evaluated by diluting aliquots of the cultures at late stationary phase (136 h) to OD_600_ 0.1, 0.01 and 0.001. 5 µl of the dilutions were inoculated on YPD-agar and incubated at 30°C for 48 h.

### Mutagenesis

Gene deletions were carried out by a PCR-based deletion strategy, as previously described [63]. Within the present study 16 genes, coding for phospholipases B (*PLB1, PLB2, PLB3* and *NTE1*), TAG synthases and lipases (*LRO1, DGA1, TGL2, TGL3, TGL4* and *TGL5*), SE synthases and hydrolases (*ARE1, ARE2, TGL1, YEH1* and *YEH2*) and an acyl-CoA synthetase (*FAT1*), were targeted for deletion in various combinations. These genes were deleted in the strain YB526 already deficient of all four FAA genes [47]. The deletion cassettes employed were hygromycin phosphotransferase (HYG) under control of the TEF promoter and the CYC terminator, nourseothricin N-acetyltransferase (NAT) under control of TEF promoter and ADH terminator, aminoglycoside 3′-phosphotransferase (KanMX4) under control of the TEF promoter and the TEF terminator, orotidine 5-phosphate decarboxylase (URA3) of *Klyveromyces lactis* driven by its native promoter and terminator and D-serine deaminase (DsdAMX4) under control of the TEF promoter and the TEF terminator. The primers used for synthesis of the deletion cassettes are described in Table S3. Plasmids carrying the cassettes templates were obtained from EUROSCARF and modified as previously described [64]. The deletion cassettes were transformed into the cells using a modified version of the protocol described previously [65], [66].

The NAT and, where indicated, the KanMX4 cassettes included locus of X-over P1 (*loxP*) sites to allow for deletion of the resistance marker and its reutilization as described before [67], [68]. In brief, after replacement of a target ORF by a *loxP* flanked marker, a pSH47 vector, selectable by uracil auxotrophy complementation and carrying the ORF of the Cre recombinase under *GAL1* control, was transformed into the cells. Expression of the recombinase was induced by galactose supplementation leading to the excision of the resistance marker. The cells were then transferred to 5-FOA plates to select for the loss of the pSH47 vector [69].

### Preparation of yeast cells and extracellular fraction

Yeast cells suspension (2 mL) was collected at the end of exponential phase (35 h) or at late stationary phase (136 h) for total FA analysis. Cells and extracellular fraction were separated by centrifugation at 1500 g for 3 min. Extracellular fraction (1 mL) was transferred to a new 2 mL microcentrifuge tube. The remainder of the supernatant was discarded. The cell pellet was washed once with water. For FA analysis in specific lipid classes 20 mL cells suspension was collected at late stationary phase (136 h). The suspension was centrifuged at 800 g for 3 min and the supernatant was discarded.

### Lipid analytical methods

Yeast cell pellet and supernatant were transferred to 10 mL glass tubes with glass-stop corks. The cell pellet was resuspended in 1 mL of distilled water and glass beads (425–600 µm) were added to break the cells. Intracellular and extracellular lipid extractions were performed using heptadecanoic acid (17∶0) as internal standard for free fatty acids (5 µg) and triheptadecanoylglycerol as internal standard for esterified fatty acids (10 µg) [70]. Samples for total FA analysis were directly subjected to derivatization as described ahead. Samples for analysis of FA in specific lipid classes were subjected to separation by TLC prior to derivatization: Samples (30% of extract derived from 20 mL cell suspension) were applied on 20×20 cm silica gel 60 plates (Merck) and resolved in a single dimension using hexane/diethyl ether/acetic acid (80∶20∶1, v/v) for neutral lipids and chloroform/methanol/acetic acid (65∶25∶ 8, v/v) for phospholipids. Lipid standards were included on each TLC plate. The plates were sprayed with ammonium 8-anilino-1-naphthalenesulfonate 0.2% (w/v) in methanol, bands were visualized under UV light and marked with a graphite pencil. Bands corresponding to PC, PE, TAG, DAG and SE were scraped from the plates and collected in 2 mL microcentrifuge tubes. 4 µg tripentadecanoylglycerol were added to each tube as secondary standard.

Free fatty acids from intracellular and extracellular lipid extracts were methylated according to a modified protocol described previously [71]. In brief, the lipid extract (50 µL) was transferred to a new glass tube and dried under a stream of nitrogen. Methanol (400 µL) was added together with 10 µL of 1-ethyl-3-(3-dimethylaminopropylcarbodiimide) (0.1 mg•L^−1^ in methanol) and incubated for 2 h at 22°C. The reaction was stopped by adding 200 µL of saturated NaCl solution. The methyl esters of free fatty acids were extracted with 1 mL of hexane followed by centrifugation at 200 g for 2 min. The upper hexane phase was transferred to a 1.5 mL microcentrifuge tube, dried, resuspended in acetonitrile (16 µL) and analyzed by gas chromatography. Esterified fatty acids recovered after TLC separation were transmethylated according to a modified protocol described previously [72]. 333 µL methanol/toluene (1∶1, v/v) and 167 µL 0.5 M sodium methoxide in methanol were added and left at 22°C. After 20 min the reaction was stopped by adding 500 µL of 1 M NaCl and 50 µL of 32% hydrochloric acid. The methyl esters of fatty acids were extracted with 1 mL of hexane followed by centrifugation at 2300 g for 2 min. The upper hexane phase was transferred to a new 1.5 mL microcentrifuge tube, dried and resuspended in acetonitrile (16 µL). The fatty acid methyl esters were analyzed by gas chromatography using an Agilent 6890 series gas chromatograph equipped with a capillary DB-23 column (Agilent Technologies, Waldbronn, Germany).

## Supporting Information

Figure S1 **Ratio of cell density to optical density is independent of genotype.** Cells from a representative subset of the strains used throughout this work were grown to late stationary phase (136 h) in YPR. Independency between the ratio and the genetic composition of the strains validates the use of OD_600_ as an indicator of cell number.
(TIF)

Figure S2 **Cell viability after stationary phase.** Cells of various mutant strains were grown to late stationary phase (136 h) in YPR. Aliquots of the cultures were diluted to OD_600_ of 0.1, 0.01 and 0.001 with sterile water. 5 µl of the dilutions where inoculated on YPD-agar plates and incubated at 30°C for 48 h.
(TIF)

Table S1 **Growth curves.**
(DOCX)

Table S2 **Composition (in percentage) of the free fatty acid pool.**
(DOC)

Table S3 **Primers for synthesis of deletion cassettes.**
(DOCX)
