# Supplementary figures and images for: Neutral Lipid Metabolism Influences Phospholipid Synthesis and Deacylation in Saccharomyces cerevisiae
Source: PLoS One. 2012 Nov 5;7(11):e49269. doi: 10.1371/journal.pone.0049269 (PMC3489728; doi:10.1371/journal.pone.0049269)

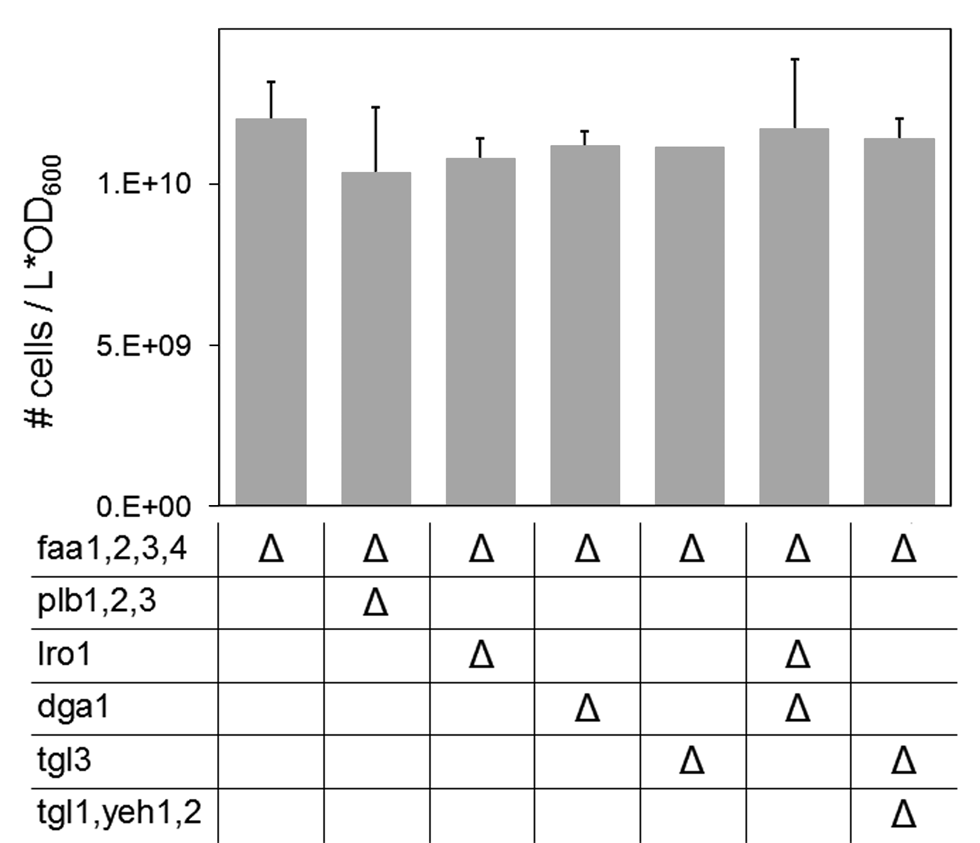

Supplement: Figure S1 — Ratio of cell density to optical density is independent of genotype. Cells from a representative subset of the strains used throughout this work were grown to late stationary phase (136 h) in YPR. Independency between the ratio and the genetic composition of the strains validates the use of OD600 as an indicator of cell number. (TIF) [file pone.0049269.s001.tif]

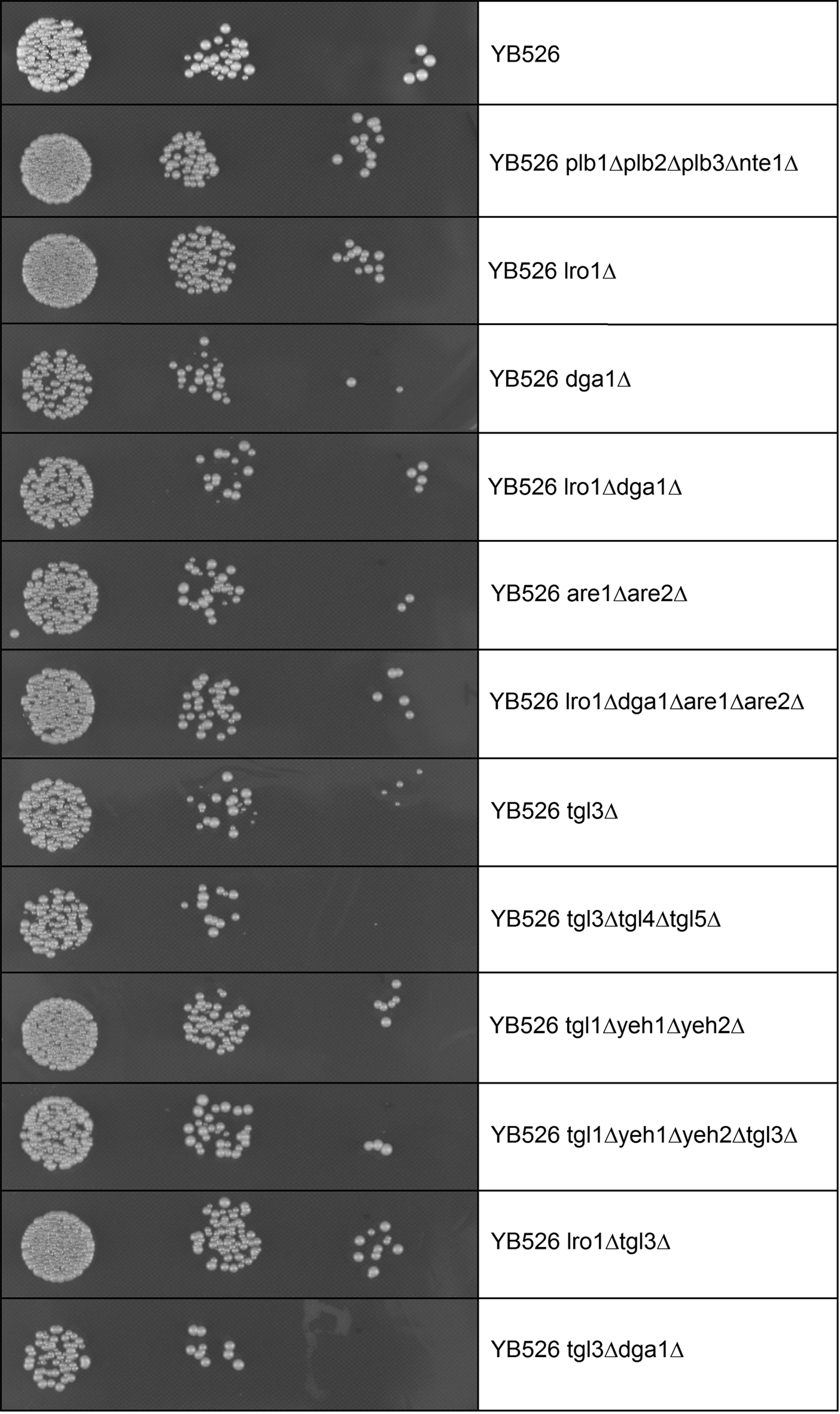

Supplement: Figure S2 — Cell viability after stationary phase. Cells of various mutant strains were grown to late stationary phase (136 h) in YPR. Aliquots of the cultures were diluted to OD600 of 0.1, 0.01 and 0.001 with sterile water. 5 µl of the dilutions where inoculated on YPD-agar plates and incubated at 30°C for 48 h. (TIF) [file pone.0049269.s002.tif]
