# Supplementary material for: Neutral Lipid Metabolism Influences Phospholipid Synthesis and Deacylation in Saccharomyces cerevisiae
Source: PLoS One. 2012 Nov 5;7(11):e49269. doi: 10.1371/journal.pone.0049269 (PMC3489728; doi:10.1371/journal.pone.0049269)
Supplement: Table S1 — Growth curves. (DOCX) [file pone.0049269.s003.docx]

**Table S1. Growth curves.**

| **Time (h)** | 0 | 18 | 24 | 40 | 46 | 63 | 69 | 92 | 136 |
| --- | --- | --- | --- | --- | --- | --- | --- | --- | --- |
| **Strain** | **Optical density at 600 nm** | | | | | | | | |
| YB526 | 0.03 | 0.53 (0.13) | 2.04 (0.60) | 6.79 (0.24) | 6.86 (0.44) | 7.72 (0.19) | 7.49 (0.27) | 7.39 (0.32) | 6.90 (0.16) |
| YB526 *plb1∆plb2∆plb3∆nte1∆* | 0.03 | 0.68 (0.12) | 2.72 (0.31) | 7.17 (0.06) | 7.29 (0.30) | 7.71 (0.26) | 7.94 (0.16) | 7.33 (0.09) | 6.70 (0.19) |
| YB526 *lro1∆* | 0.03 | 0.88 (0.07) | 3.12 (0.08) | 6.71 (0.16) | 7.23 (0.54) | 7.83 (0.12) | 7.65 (0.02) | 7.12 (0.11) | 6.60 (0.29) |
| YB526 *dga1∆* | 0.03 | 0.41 (0.05) | 1.46 (0.28) | 6.19 (0.30) | 6.17 (0.23) | 7.01 (0.19) | 6.79 (0.23) | 7.15 (0.20) | 6.70 (0.19) |
| YB526 *lro1∆dga1∆* | 0.03 | 0.74 (0.08) | 2.68 (0.18) | 6.27 (0.20) | 6.61 (0.25) | 7.36 (0.45) | 7.37 (0.17) | 6.79 (0.16) | 6.00 (0.14) |
| YB526 *are1∆are2∆* | 0.03 | 0.46 (0.17) | 1.66 (0.79) | 6.75 (0.49) | 6.79 (0.10) | 7.70 (0.44) | 7.65 (0.31) | 7.68 (0.13) | 7.20 (0.16) |
| YB526 *lro1∆dga1∆are1∆are2∆* | 0.03 | 0.55 (0.04) | 1.97 (0.19) | 6.18 (0.24) | 6.39 (0.18) | 7.20 (0.45) | 7.37 (0.11) | 6.26 (0.33) | 5.90 (0.05) |
| YB526 *tgl3∆* | 0.03 | 0.47 (0.02) | 1.78 (0.04) | 6.65 (0.18) | 7.09 (0.25) | 8.04 (0.26) | 8.09 (0.43) | 7.87 (0.06) | 7.27 (0.06) |
| YB526 *tgl3∆tgl4∆tgl5∆* | 0.03 | 0.38 (0.03) | 1.36 (0.17) | 6.81 (0.10) | 7.17 (0.12) | 7.85 (0.21) | 8.12 (0.26) | 7.81 (0.13) | 7.20 (0.03) |
| YB526 *tgl1∆yeh1∆yeh2∆* | 0.03 | 0.55 (0.04) | 2.10 (0.15) | 7.40 (0.19) | 7.45 (0.22) | 8.23 (0.20) | 8.01 (0.24) | 7.56 (0.14) | 7.03 (0.16) |
| YB526 *tgl1∆yeh1∆yeh2∆tgl3∆* | 0.03 | 0.72 (0.09) | 2.87 (0.27) | 7.30 (0.29) | 7.29 (0.29) | 8.34 (0.03) | 8.15 (0.38) | 7.61 (0.15) | 7.08 (0.07) |
| YB526 *lro1∆tgl3∆* | 0.03 | 0.50 (0.03) | 2.05 (0.17) | 7.31 (0.03) | 7.63 (0.25) | 7.85 (0.29) | 7.79 (0.35) | 7.40 (0.22) | 6.89 (0.21) |
| YB526 *tgl3∆dga1∆* | 0.03 | 0.50 (0.05) | 2.00 (0.21) | 6.63 (0.30) | 6.91 (0.19) | 7.45 (0.25) | 7.81 (0.39) | 7.44 (0.12) | 6.45 (0.24) |

Cells of the various mutant strains were grown for 18 h in YPR and diluted in YPR until OD_600_ of 0.03 was reached. The cells were kept at 30 °C with permanent agitation (210 rpm) and the evolution of the cultures was monitored by OD_600_. Mean values of three independent experiments; standard deviation is shown within parentheses.
