# Supplementary material for: Neutral Lipid Metabolism Influences Phospholipid Synthesis and Deacylation in Saccharomyces cerevisiae
Source: PLoS One. 2012 Nov 5;7(11):e49269. doi: 10.1371/journal.pone.0049269 (PMC3489728; doi:10.1371/journal.pone.0049269)
Supplement: Table S2 — Composition (in percentage) of the free fatty acid pool. (DOC) [file pone.0049269.s004.doc]

**Table S2. Composition (in percentage) of the free fatty acid pool.**

| **A (136h)** | 16:0 | 16:1 | 18:0 | 18:1 | Total FA | |
| --- | --- | --- | --- | --- | --- | --- |
| strain | % | | | | | µmol/L∙OD |
| YB332 | 18 (3.1) | 45 (8.3) | 12 (3.5) | 25 (2.2) | 0.8 | |
| YB526 | 30 (2.5) | 26 (1.6) | 13 (0.7) | 28 (1.8) | 69.9 | |
| YB526*plb1Δ* | 33 (0.5) | 26 (0.6) | 13 (0.2) | 26 (0.2) | 76.4 | |
| YB526*plb2Δ* | 31 (0.5) | 27 (0.2) | 13 (0.2) | 27 (0.6) | 75.1 | |
| YB526*plb3Δ* | 31 (0.7) | 27 (0.7) | 12 (0.9) | 27 (0.7) | 71.0 | |
| YB526*nte1Δ* | 31 (1.0) | 25 (0.5) | 14 (0.2) | 28 (1.0) | 74.9 | |
| YB526*plb1Δplb2Δplb3Δ* | 35 (0.3) | 25 (0.2) | 13 (0.1) | 24 (0.3) | 62.8 | |
| YB526*plb1Δplb2Δplb3Δnte1Δ* | 35 (1.7) | 22 (0.9) | 17 (0.6) | 24 (1.2) | 66.4 | |
| YB526*tgl2Δ* | 30 (1.1) | 27 (0.8) | 13 (0.2) | 28 (0.7) | 65.2 | |
| YB526*tgl3Δ* | 31 (5.9) | 25 (2.7) | 13 (0.3) | 28 (3.5) | 53.8 | |
| YB526*tgl4Δ* | 28 (4.0) | 27 (1.7) | 13 (0.3) | 30 (2.7) | 64.9 | |
| YB526*tgl5Δ* | 30 (4.4) | 26 (2.4) | 12 (0.4) | 28 (2.5) | 73.5 | |
| YB526*tgl3Δtgl4Δtgl5Δ* | 29 (0.2) | 28 (0.5) | 12 (0.4) | 28 (0.5) | 54.2 | |
| YB526*lro1Δ* | 30 (1.1) | 29 (1.3) | 11 (0.5) | 27 (0.4) | 50.3 | |
| YB526*lro1Δtgl3Δ* | 35 (0.8) | 25 (0.5) | 12 (0.2) | 25 (0.6) | 50.2 | |
| YB526*dga1Δ* | 32 (0.8) | 26 (0.6) | 13 (0.3) | 26 (0.5) | 69.0 | |
| YB526*tgl3Δdga1Δ* | 33 (0.6) | 26 (0.5) | 13 (0.2) | 26 (0.3) | 65.7 | |
| YB526*lro1Δdga1Δ* | 32 (0.4) | 27 (0.3) | 12 (0.2) | 26 (0.2) | 65.1 | |
| YB526*lro1Δnte1Δ* | 31 (1.2) | 27 (1.0) | 13 (0.5) | 26 (1.0) | 52.6 | |
| YB526*nte1Δdga1Δ* | 32 (0.3) | 25 (0.5) | 14 (0.4) | 26 (0.1) | 61.0 | |
| YB526*lro1Δnte1Δdga1Δ* | 32 (0.9) | 27 (0.8) | 13 (0.4) | 26 (0.6) | 59.4 | |
| YB526 *plb1Δplb2Δplb3Δnte1Δdga1Δ* | 39 (0.5) | 22 (0.6) | 11 (0.3) | 26 (0.2) | 61.2 | |
| YB526*are1Δare2Δ* | 31 (1.4) | 26 (1.1) | 14 (0.8) | 26 (1.2) | 47.8 | |
| YB526*lro1Δdga1Δare1Δare2Δ* | 28 (1.1) | 26 (1.2) | 13 (0.9) | 29 (0.8) | 66.1 | |
| YB526*yeh1Δ* | 36 (1.5) | 23 (1.0) | 14 (0.3) | 24 (0.8) | 68.5 | |
| YB526*yeh2Δ* | 35 (1.1) | 23 (0.2) | 14 (0.3) | 25 (0.8) | 61.9 | |
| YB526*tgl1Δ* | 35 (3.9) | 24 (2.3) | 14 (0.2) | 25 (2.1) | 65.1 | |
| YB526*tgl1Δyeh1Δyeh2Δ* | 34 (1.7) | 26 (0.5) | 13 (0.4) | 25 (0.9) | 52.3 | |
| YB526*tgl1Δyeh1Δyeh2Δtgl3Δ* | 36 (1.3) | 25 (1.2) | 12 (0.5) | 24 (0.6) | 40.5 | |
| YB526*fat1Δ* | 28 (0.4) | 35 (0.6) | 8 (0.3) | 26 (0.3) | 83.4 | |
| YB526*lro1Δ fat1Δ* | 32 (0.6) | 29 (0.7) | 11 (0.4) | 26 (0.3) | 73.1 | |
| YB526*dga1Δ fat1Δ* | 33 (2.3) | 30 (1.8) | 11 (0.4) | 23 (1.0) | 81.6 | |
| YB526*lro1Δdga1Δ fat1Δ* | 34 (1.1) | 28 (0.5) | 11 (0.3) | 24 (1.1) | 84.9 | |
| YB526*tgl3Δ fat1Δ* | 36 (1.8) | 28 (1.4) | 11 (0.3) | 23 (0.8) | 66.1 | |

| **B (35 h)** | 16:0 | 16:1 | 18:0 | 18:1 | Total FA | |
| --- | --- | --- | --- | --- | --- | --- |
| strain | % | | | | | µmol/L∙OD |
| YB526 | 38 (0.0) | 25 (0.1) | 12 (0.4) | 22 (0.5) | 34.2 | |
| YB526*plb1Δplb2Δplb3nte1Δ* | 38 (0.3) | 25 (0.3) | 13 (0.1) | 21 (0.2) | 31.7 | |
| YB526*lro1Δ* | 35 (0.2) | 27 (0.3) | 13 (0.1) | 23 (0.0) | 37.3 | |
| YB526*tgl3Δ* | 41 (0.2) | 24 (0.2) | 12 (0.2) | 20 (0.3) | 34.3 | |
| YB526*lro1Δtgl3Δ* | 38 (0.2) | 25 (0.2) | 13 (0.0) | 21 (0.1) | 33.0 | |
| YB526*lro1Δdga1Δ* | 35 (0.2) | 26 (0.3) | 14 (0.2) | 22 (0.1) | 40.2 | |

Cells were grown to **(A)** late stationary phase (136 h) and **(B)** end of exponential phase (35 h) in YPR. Lipid extracts were methylated and analyzed by GC. The values reported correspond to total FFA (cells and medium). 14:0 is included in the total but is not presented in the table. Mean values of at least three independent experiments. Standard deviation is shown within parentheses.
