# Supplementary material for: Neutral Lipid Metabolism Influences Phospholipid Synthesis and Deacylation in Saccharomyces cerevisiae
Source: PLoS One. 2012 Nov 5;7(11):e49269. doi: 10.1371/journal.pone.0049269 (PMC3489728; doi:10.1371/journal.pone.0049269)
Supplement: Table S3 — Primers for synthesis of deletion cassettes. (DOCX) [file pone.0049269.s005.docx]

**Table S3. Primers for synthesis of deletion cassettes.**

| ARE1UP: | TCCGCAGACTCAATTCCGCAGAAGCCAACAAACGGCATTCGGTCACGTACGCTGCAGGTCGAC |
| --- | --- |
| ARE1DO: | TCTTTGACCACGGTGGAGCGGACGCCCATTCCCTATTGTATATCTATCGATGAATTCGAGCTCG |
| ARE2UP: | CCAAAGAGAAGGCACGGTATAGGCAAGGGTCCTCTAACTTTATATCGTACGCTGCAGGTCGAC |
| ARE2DO: | TGCAGCATTTGGAAGAAGAACAAGTAAAACCTCAATTTCTTGAAGATCGATGAATTCGAGCTCG |
| DGA1UP: | CTGTAGCATGGCACACTTCTTCATTTGTACTCTTCTCCATATTTACGTACGCTGCAGGTCGAC |
| DGA1DO: | TCCAACAACAACATTGATAGGCGCTCTAAATGGCAACAAACCGAAATCGATGAATTCGAGCTCG |
| FAT1UP: | ATTCTATATCGTTGAACTTTTAATAGGCTGCGAATACCGACTATGCGTACGCTGCAGGTCGAC |
| FAT1DO: | CATCCAAACCCTTTGGTAATTTTTGCTCTCTATAAACCTTCTTCAATCGATGAATTCGAGCTCG |
| LRO1UP: | CGTGGCAAAGATTTCGACAGGAAAAGAGACGGGAACGGTAGAAAACGTACGCTGCAGGTCGAC |
| LRO1DO: | GGATGTCTACGTGTTCGGCGCTTTTTGCTCCACCACGTATATCAAATCGATGAATTCGAGCTCG |
| NTE1UP: | TTCAACTTCTCGTGGGTTGTGTCCTACTTTGTTATGGGTGCCTCTCGTACGCTGCAGGTC |
| NTE1DO: | ACCAAATCATAATCCTTCGCGTACAAACCGCCAACAAAGGAACCAATCGATGAATTCGAG |
| PLB1UP: | GAACGTTAACGCTTGGTCACCAAATAACAGTTACGTCCCTGCGAACGTACGCTGCAGGTCGAC |
| PLB1DO: | GAAGAATAATCATCATTTCCTACACCTGAGACTGACCTGCTGTCAATCGATGAATTCGAGCTCG |
| PLB2UP: | TACAGGCTAGCTCGCTAATTTCTGGACTTTCGCTCGCTGCAGATTCGTACGCTGCAGGTCGAC |
| PLB2DO: | GAACCTAAGAGAGCCGTTATTGGAATACCGTCAGTAGCCTCAGAGATCGATGAATTCGAGCTCG |
| PLB3UP: | TCAGTTACATATTCGCAATTTCTCAGTTTCTACTGGCCGCTAATGCGTACGCTGCAGGTCGAC |
| PLB3DO: | GCATAATCATAGCAGAGAACTTTACACTTATGCCAGATAAATGTGATCGATGAATTCGAGCTCG |
| TGL1UP: | TTATTATTCTAGCACTATTTTAAAAAACTGTCTTTTGGCAAAATGCGTACGCTGCAGGTCGAC |
| TGL1DO: | CTAGACAAAAAATAGTTTAATAGGGTTTCTCTCGCATTCTTTTCAATCGATGAATTCGAGCTCG |
| TGL2UP: | AATCCCTTCTGTATTCCATCTGACAAACCTAATTTCCAATTCAATCGTACGCTGCAGGTCGAC |
| TGL2DO: | TTGGCGTAACCAAATTGAAATATTTCATATACGCAGTCGTGAGTTATCGATGAATTCGAGCTCG |
| TGL3UP: | GTTAGATGAGTGTGATACGTATCAAATGTGGTGTCAGCAAGCGTCCGTACGCTGCAGGTCGAC |
| TGL3DO: | TTCAACTGCGGTATGCCCAATTCTGGGATTATGTACTCTGCTGAAATCGATGAATTCGAGCTCG |
| TGL4UP: | GCAAGGGTCATCTTTAGCCAGTAAATGCAAATCATTTCTTTACAACGTACGCTGCAGGTCGAC |
| TGL4DO: | ACGCCTGAAATTGCCCGCTAAATCACTATGAATCATTGCGTCTGAATCGATGAATTCGAGCTCG |
| TGL5UP: | GAGTCCTCGTTGTATAACTATAAGCTGCTGCAAGACTTGACCATCCGTACGCTGCAGGTCGAC |
| TGL5DO: | GCAAGTCAGCATCCATTATGGAAAGAGATTTGATTTGCTCACTGAATCGATGAATTCGAGCTCG |
| YEH1UP: | AATAGTTTTATATATAGGTATATTTACTGCACAATTCACACGATGCGTACGCTGCAGGTCGAC |
| YEH1DO: | CCAGATCTTGTAGTTGACATTCGACTCCACATTGACGAAATGATTATCGATGAATTCGAGCTCG |
| YEH2UP: | AACGCCCTTAGATTTACAGCGGGATCAGGAAAATAATATTGAATACGTACGCTGCAGGTCGAC |
| YEH2DO: | TATTATATTTTACAAAGAAACCACAAAGAAAAAACTTTTACCTCAATCGATGAATTCGAGCTC |
